# Esophageal cancer practice guidelines 2017 edited by the Japan Esophageal Society: part 1

**DOI:** 10.1007/s10388-018-0641-9

**Published:** 2018-08-31

**Authors:** Yuko Kitagawa, Takashi Uno, Tsuneo Oyama, Ken Kato, Hiroyuki Kato, Hirofumi Kawakubo, Osamu Kawamura, Motoyasu Kusano, Hiroyuki Kuwano, Hiroya Takeuchi, Yasushi Toh, Yuichiro Doki, Yoshio Naomoto, Kenji Nemoto, Eisuke Booka, Hisahiro Matsubara, Tatsuya Miyazaki, Manabu Muto, Akio Yanagisawa, Masahiro Yoshida

**Affiliations:** 1 Department of Surgery, Keio University School of Medicine, 35 Shinanomachi, Shinjuku-ku, Tokyo, 160-8582 Japan; 2 Department of Radiology, Graduate School of Medicine, Chiba University, Chiba, Japan; 3 Department of Gastroenterology, Saku Central Hospital, Nagano, Japan; 4 Gastrointestinal Medical Oncology Division, National Cancer Center Hospital, Tokyo, Japan; 5 Department of Gastrointestinal Tract Surgery, Fukushima Medical University School of Medicine, Fukushima, Japan; 6 Department of Endoscopy and Endoscopic Surgery, Gunma University Hospital, Maebashi, Gunma Japan; 7 Department of General Surgical Science, Gunma University Graduate School of Medicine, Maebashi, Gunma Japan; 8 Department of Surgery, Hamamatsu University School of Medicine, Hamamatsu, Shizuoka Japan; 9 Department of Gastroenterological Surgery, National Kyushu Cancer Center, Fukuoka, Japan; 10 Department of Gastroenterological Surgery, Osaka University Graduate School of Medicine, Suita, Osaka Japan; 11 Department of General Surgery, Kawasaki Medical School, Okayama, Japan; 12 Department of Radiation Oncology, Yamagata University School of Medicine, Yonezawa, Japan; 13 Department of Frontier Surgery, Graduate School of Medicine, Chiba University, Chiba, Japan; 14 Department of Clinical Oncology, Kyoto University Hospital, Kyoto, Japan; 15 Department of Pathology, Kyoto Prefectural University of Medicine, Kyoto, Japan; 16 Department of Hemodialysis and Surgery, Chemotherapy Research Institute, International University of Health and Welfare, Ichikawa, Japan

**Keywords:** Practice guidelines, Esophagus, Cancer

## Introduction

### Purpose of the guidelines

The primary objective of these guidelines is to provide general clinicians with information that would guide them to make informed choices of the available diagnosis/treatment strategies for esophageal cancer (intended for malignant esophageal tumors of epithelial origin, not for any other non-epithelial malignant tumors of the esophagus or metastatic esophageal malignant tumors). Furthermore, these guidelines are also intended as an aid for healthcare professionals other than the physicians, patients, and patients’ family members, to obtain an understanding of the fundamental principles of the diagnosis and treatment of esophageal cancer. These guidelines are intended to allow physicians to undertake diagnosis and treatment of esophageal cancer by sharing the information contained in the guidelines and promote mutual understanding among the healthcare professionals, patients, and their family members.

### Method of development of the esophageal cancer practice guideline

#### Scope formulation

The present revision of the guidelines was carried out based on the following.

1. Basic principles adopted for the preparation of the guidelines.

The basic principles for developing the 4th edition were deliberated on at the meeting of the 1st Committee on Guidelines for Diagnosis and Treatment of Esophageal Cancer in June 2012. With the intention to starting with the present Edition, a more detailed algorithm for each stage of the disease was prepared, in addition to the algorithm providing a bird’s eye view of the entire flow of diagnosis and treatment of esophageal cancer. Clinical Questions (CQs) relating to diverging points of the algorithm that would require judgment in the clinical practice setting were to be extracted.

(2) Major changes in the guidelines resulting from this revision.
  By-stage algorithms have been introduced.
  Revisions of the Guidelines so that they are readily comprehensible not only to healthcare professionals, but also to individuals on the side of the patient were attempted via modification of expressions in the text.
  More emphasis is placed on thoracoscopic esophagectomy, which is now widely used.
(3) On the methodology of preparation of the guidelines.

The guidelines were prepared by referring to the “Guide to Preparation of Guidelines for Diagnosis and Treatment 2014,” issued by Information Division of the Medical Information Network Distribution Service EBM (Minds), the Japan Council for Quality Health Care.

#### Preparation of CQs and search of the literature

The 77 CQs contained in the 3rd Edition of the guidelines were reexamined to screen out those which were not considered as important for clinical judgments concerning the therapeutic outcomes or for clinical judgment in relation with diverging points on the algorithms for diagnosis and treatment. The Japan Medical Library Association was entrusted with a systematic search of the literature published from January 1995 through June 2016, using key words extracted from the CQs. PubMed and Cochrane Library were used for the search of articles in the English language, and ICHUSHI-Web for articles published in Japanese.

Concrete key words and results of the search of the literature are described in the detailed version (website of the Japan Esophageal Society: https://www.esophagus.jp/).

Moreover, articles/papers that had escaped retrieval by the systematic search were also additionally searched for, as needed, on the ground of information provided by the systematic review team and the Preparation Committee members.

1. Inclusion criteria.

Randomized comparative studies and observational researches among studies in adult patients with esophageal cancer were adopted in principle. Studies on accumulated cases, nevertheless, were also actively adopted, depending on the outcomes determined. Only papers written in Japanese or English were adopted. Contents of other documents, such as expertise reviews and guidelines from other countries, were also reviewed in detail as reference data, although none of these was used as evidence.

(2) Exclusion criteria.

Genetic studies and experimental studies in laboratory animals were excluded.

#### Systematic review procedure

For each of the CQs, the outcomes as to the balance between the benefits and risks were extracted and the level of importance thereof was presented. Each retrieved article was subjected to a primary and a secondary screening, summarized, and then assessed for bias, besides classification of the study design (Table 1). For each outcome as to the benefits and risks, individual papers were summed up and evaluated as “a whole body of evidence” (Tables 2, 3A–D). Evaluation of the information as a “whole body of evidence” was carried out by referring to the GRADE system (Table 2). The “whole body of evidence for individual outcomes” was then summated to determine and state the quality of evidence as a whole for each CQ (Table 3).

**Table 1:** Bias risk assessment items

Selection bias	
(1)	Random sequence generation
	Is there a detailed description of whether the patient allocation was randomized?
(2)	Concealment
	Whether the person in charge of patient inclusion was concealed from the allocation status of the included patients
	(Whether the operations of randomization were isolated and independent from the clinical practice site and centralized)
Action bias	
(3)	Blinding
	Whether the study subjects were blinded, and the caregivers were blinded?
	(Whether neither the subjects nor the healthcare professionals knew which subjects were allocated to either group)
Detection bias	
(4)	Blinding
	Whether the outcome evaluator was blinded
Case decrease bias	
(5)	Intention-to-treat (ITT) analysis
	Citing the principles of ITT analysis, whether those principles were followed for dropouts from the follow-up
	(Dropouts and subjects lost to follow-up should not be excluded, but counted as “no effect” or “no response”)
(6)	Incomplete outcome data
	Whether the data reported on the respective major outcomes complete.
	(Inclusive of data adopted for and precluded from the analysis)
Other biases	
Selective outcomes reporting	
Whether there is any unreported outcome besides the outcome stated in the protocol	
Early termination of study	
Whether the study was prematurely terminated on account of achieved/anticipated benefits	
Other biases

**Table 2:** Overall evaluation of the collected articles for each outcome and each study design

(1)	Initial evaluation: evaluation for each study design group	
	SR (systematic review), MA (meta-analysis), RCT (randomized controlled trial) group	“Initial evaluation A”
	OS (observational study) group	“Initial evaluation C”
	CS (case accumulation, case report) group	“Initial evaluation D”
(2)	Assessment as to the presence of any lowering the level of evidence	
	Presence of bias risks on the quality of the study (Results of Table 1)	
	Results are inconsistent	Varying results among papers
	Evidence is indirect	There are discrepancies between the contents of the papers and the CQs. Or, the contents of papers cannot be directly applied to the clinical setting in Japan (e.g., practices covered by the national health insurance)
	Data are inaccurate	Number of cases is insufficient or does not reach the anticipated level
	High possibility of publication bias	Only favorable results are reported
(3)	Assessment as to the presence of any factors elevating the level of evidence	
	Noticeably effective, with no involvement of confounding factors	Marked efficacy may be expected in all patients
	Presence of a dose–response gradient	Further response may be anticipated with dose elevation
	Possible confounding factors underestimating the true effect	
Overall evaluation: Final certainty of the evidence was classified as “A, B, C, or D.”

**Table 3:** Overall evaluation of the collected articles for each outcome and each study design

A	High-quality evidence (High)
	We are very confident that the true effect lies close to the estimated effect
B	Moderate-quality evidence (Moderate)
	We are moderately confident about the estimated effect
	The true effect is likely to be close to the estimated effect, but there is a possibility that it is substantially different
C	Low-quality evidence (Low)
	Our confidence in the estimated effect is limited
	The true effect may be substantially different from the estimated effect
D	Very low-quality evidence (Very Low)
	We have very little confidence in the estimated effect
	The true effect is likely to be substantially different from the estimated effect

#### Determination of the strength of recommendations

The members of the Guideline Preparation Committee prepared drafts of our recommendation statements based on the results of a systematic review, and a consensus conference was held to examine the strength of the recommendations. The strength of each recommendation was examined on the ground of certainty of evidence, benefits and risks, patient preferences, and cost evaluation. As for the method of arriving at a consensus, a secret ballot was held with independent voting by 20 members of the Guideline Preparation Committee using an Answer Pad in accordance with the modified Delphi method and nominal group technique; the strength of the recommendation was determined based on a ≥ 70% consensus. When a ≥ 70% consensus was not achieved in the first vote, a second vote was called for after consultation. In the case failure to arrive at a consensus even on the second vote, it was stated that the strength of recommendation could not be determined.

The strength of recommendation was expressed in 2 directions × 2 steps as follows:

1. Conduct or non-conduct is “strongly recommended.”
2. Conduct or non-conduct is “weakly recommended.”

### Conflict of interest (COI) and economic independence

#### Conflict-of-interest (COI) reporting

Members of the Guideline Review Committee and Guideline Steering Committee personally reported their conflicts of interests in conformity with the regulations of the Japan Esophageal Society. The Ethics Committee and the Board of Directors of the Japan Esophageal Society confirmed the personally reported conflict-of-interest situations. Situations of conflicts of interests are uploaded on the website of the Japan Esophageal Society for each fiscal year.

#### Restrictions at the recommendation decision conference based on COI

In the case that any member of the Guideline Steering Committee (1) is an author of a paper serving as a basis for preparation of these guidelines (academic COI) or (2) has a COI concerning an enterprise or competing enterprise pertaining to manufacture and/or marketing of a related drug(s) or medical device(s) (economical COI), the said member will not participate in the voting at the consensus conference by self-declaration.

#### Efforts to prevent academic bias unique to the society

Efforts were made to avoid academic COI of any single academic organization by constructing a cooperative system with a plurality of related academic bodies.

#### Economic independence

The Japan Esophageal Society met the expenditure for the preparation and publication of these guidelines and has not received any funding from enterprises.

## Epidemiology, present status, and risk factors

### Summary

As for the dynamic trends of esophageal carcinoma in Japan, the incidence rate has been gradually rising in men, while essentially remaining constant in women. The mortality rate has been leveling off in men, whereas it has been decreasing in women. Among patients with this malignancy, the percentage of males is higher, as is the percentage of patients in their 60–70s. The carcinoma is most frequently located in the middle thoracic esophagus. Squamous cell carcinoma is the predominant histologic type, accounting for about 90% of all cases. Esophageal cancer is known to be frequently associated with synchronous or metachronous multiple carcinoma. The risk factors cited for esophageal squamous cell carcinoma include smoking and habitual alcohol consumption. As regards the risk factors for adenocarcinoma, Barrett’s epithelium arising from persistent inflammation of the lower esophagus due to gastroesophageal reflux disease (GERD) has been reported to serve as a risk factor for the development of esophageal carcinoma in Europe and the United States. In Japan, however, the risk associated with this factor remains unclear because of the scarcity of documented cases.

### General remarks

#### Incidence and mortality

According to the statistics released by the Center for Cancer Control and Information Services, National Cancer Center, based on the cancer incidence (morbidity incidence rate) data derived from the Population-Based Cancer Registry, the estimated incidence rate of esophageal carcinoma (crude incidence rate) in 2011 was 31.7 persons per 100,000 population in men and 5.2 persons per 100,000 population in women. The age-adjusted incidence rate has been showing a gently upward trend in men, while no appreciable upward or downward trend has been seen in women.

The vital statistics compiled by the Ministry of Health, Labour and Welfare showed that there were 11,543 deaths from esophageal carcinoma in 2013 (crude mortality rate: 9.2 persons per 100 000 population), accounting for 3.2% of all deaths from malignant neoplasms. The crude mortality rate associated with esophageal carcinoma in men was 15.8 persons per 100,000 population, ranking below the rates for cancers of the lung, stomach, large intestine, liver, and pancreas, and the rate in women was 2.9 persons (per 100,000 population), ranking below the tenth place. The age-adjusted mortality rate of esophageal carcinoma has been leveling off in men and decreasing in women. Cancer mortality data derived from vital statistics and various graphs based on those data are available at the Center for Cancer Control and Information Services, National Cancer Center (http://ganjoho.jp/reg_stat/index.html).

### Glossary

*Incidence rate* Number of cases detected in a certain population during a certain period of time divided by the number of individuals in the population. The data shown are those provided by the Center for Cancer Control and Information Services, National Cancer Center, on the basis of the cancer morbidity data derived from the Population-Based Cancer Registry (1975–2011).

*Age-adjusted incidence rate* Incidence rate that would have been observed if the age composition of the population was the same as that of the base population.

*Crude mortality rate* The number of deaths occurring during a certain period of time divided by the number of individuals in the population during the period.

*Age-adjusted mortality rate* The mortality rate that would have been observed if the age composition of the population was the same as that of the base population. Because of the increase in the cancer mortality rate with advancing age, the crude mortality rate is higher in populations containing a greater proportion of elderly people than in those containing a smaller proportion of elderly people. Therefore, the mortality rate in a population as a whole is obtained after adjusting for the age composition of a population used as the base (base population). The 1985 model population (virtual population model based on Japan’s population in 1985) is the base population used in Japan.

### Present status of esophageal carcinoma in Japan

A nationwide survey conducted by the Japan Esophageal Society (2008) [1]. According to the survey, male patients outnumber female patients, with a male–female ratio of about 6:1, and most patients are in their 60 or 70s, these age groups accounting for about 69% of the patients overall. The carcinoma is predominantly located in the middle thoracic esophagus (in approximately 50% of cases), followed, in order of frequency, by the lower thoracic esophagus (in approximately 25% of cases), upper thoracic esophagus (in approximately 12% of cases), abdominal esophagus (in approximately 6% of cases), and cervical esophagus (in approximately 5% of cases), squamous cell carcinoma is the overwhelmingly frequent histologic type, accounting for about 90% of all cases, followed in frequency by adenocarcinoma, which accounts for about 4% of all cases. About 23% of patients with esophageal carcinoma have synchronous or metachronous multiple cancer, with the most frequently observed being gastric cancer, followed in frequency by pharyngeal cancer; this is an important statistic from the point of view of the clinical diagnosis and treatment of esophageal carcinoma.

### Risk factors

The most frequent risk factors for esophageal carcinoma identified in Japan are habitual alcohol consumption and the smoking habit. These are the most important risk factors for squamous cell carcinoma, being identified as risk factors in more than 90% of all cases of esophageal carcinoma in Japan. Concomitant use of tobacco and alcohol has been shown to be associated with a multiplied risk of development of esophageal carcinoma [2–5]. In October 2009, a working group of the International Agency for Research on Cancer (IARC), a substructure of the World Health Organization, specified that the acetaldehyde formed after consumption of alcoholic beverages is a Group 1 carcinogen [5]. Besides, in relation with dietary factors, poor nutritional status and vitamin deficiencies due to inadequate intake of fruits and vegetables have also been reported as risk factors. By contrast, intake of green and yellow vegetables and fruits has been reported as preventive factors [6, 7].

While adenocarcinoma accounts for only a small percentage of patients with esophageal carcinoma, the percentage esophageal carcinoma patients with this histological subtype is increasing in Europe and North America, accounting for about more than half of all the cases of esophageal carcinoma. Barrett’s epithelium caused by persistent inflammation of the lower esophagus due to GERD is known to serve as a predisposing lesion for the development of esophageal adenocarcinoma, and there have been reports indicating that GERD, high body mass index (BMI), which serves as a risk factor for GERD, and smoking are involved in the development of it [8–11]. In Japan, however, no clear evidence has been established because of the scarcity of cases.

## Treatment algorithms for esophageal cancer and treatment policies based on the algorithm

### Japanese classification of esophageal cancer and tumor, node, metastasis (TNM) (Union for International Cancer Control [UICC]) classification

It should be noted that there exists some discordance on the subject of disease staging in these guidelines, as the disease staging was conducted in accordance with the Japanese Classification of Esophageal Cancer and the edition of the TNM (UICC) Classification prevailing at that time. However, a by-histologic-type classification system is adopted in the 8th Edition of TNM (UICC), in consideration of the difference in the prognosis between squamous cell carcinoma and adenocarcinoma, inferred largely from therapeutic outcomes reported from Europe and the United States. In the present guidelines, the by-stage treatment algorithm is based on the 11th Edition of the Japan Esophageal Society’s Japanese Classification of Esophageal Cancer.

### Treatment algorithm for cStage 0–I esophageal cancer (Fig. 1)

#### Summary

To select the treatment policy for cStage 0 or I carcinoma of the esophagus, the clinical stage of the disease should first be confirmed by such means as endoscopic examination, computed tomography (CT) of the neck, chest and abdomen, and positron-emission tomography (PET). Then, it is important to assess the depth of tumor invasion to select the most appropriate treatment from among the options of endoscopic resection (ER), surgery, and chemoradiotherapy.

**Fig. 1:**
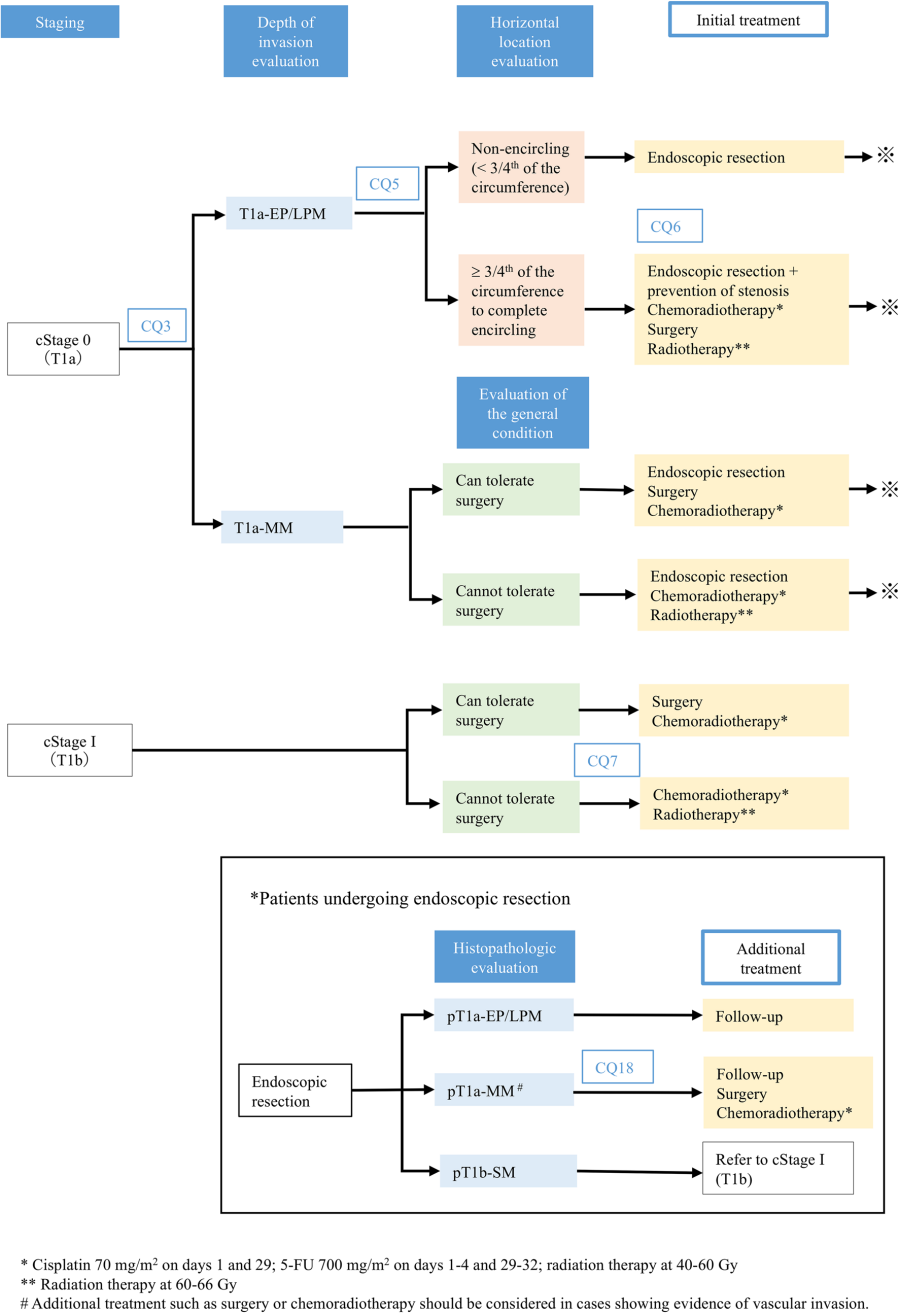
Treatment algorithms for cStage 0, I esophageal cancer

Minimally invasive ER should be considered, where the physician wavers in his/her assessment of the depth of tumor invasion and in patients in a poor general condition. Assessment of the circumferential extent of the lesion should be undertaken in patients with cStage 0 (T1a) scheduled to undergo ER for predicting the risk of development of post-ER stenosis. For a lesion involving ≥ 3/4th of the esophageal circumference, a preventive strategy against stenosis should be considered, as such lesions are associated with a high risk of development of stenosis after ER.

Post-ER histopathologic assessment is of vital importance to determine if any additional treatment is required or not. In patients classified as having pT1a-epithelium (EP)/lamina propria mucosae (LPM) disease, follow-up should be scheduled; on the other hand, in patients diagnosed as having pT1a-muscularis mucosae (MM)/pT1b-submucosal (SM) disease, additional treatment (surgery or chemoradiotherapy) should be considered. In patients with cStage I (T1b) disease, the selection between surgery and chemoradiotherapy should be made after assessing the patient’s surgical tolerability.

#### CQ3: What would be a recommended method for clinical diagnostic differentiation between T1a-EP/LPM and T1a-MM disease in patients with superficial cancer of the esophagus?

### Recommendation statement

There is weak evidence to recommend ultrasound endoscopy or magnifying endoscopy for clinical diagnostic differentiation between T1a-EP/LPM and T1a-MM disease among cases of superficial cancer of the esophagus (*Rate of consensus: 94.7% [18/19], strength of evidence: C*)

### Explanatory note

A search of the literature in relation with this CQ yielded 139 PubMed articles, 54 Cochrane articles, and 166 ICHUSHI articles, along with an additional 18 references including reviews on diagnosis of the depth of invasion, etc. Hence, a total of 377 articles were subjected to a primary screening. From these, 77 articles were subjected to a secondary screening, and finally, 13 articles [12–14] were selected for qualitative systematic reviews.

All the 13 papers were from Japan; none of them pertained to any randomized comparative trials and none were reports of any comparative study with different modalities. Of the 13 papers, 2 were related to investigating the diagnostic accuracy of non-magnifying endoscopy, 6 papers investigating that of magnifying endoscopy [13], and 4 papers investigating that of endoscopic ultrasonography (EUS) [14]. The remaining 1 paper described a study involving magnifying endoscopy performed after non-magnifying endoscopic observation [12].

Since there was no study of a direct comparison of the diagnostic procedures, comparison among the different modalities was carried out using the summary receiver operating characteristic (ROC) curve. This comparison demonstrated a higher diagnostic accuracy of EUS and magnifying endoscopy than that of non-magnifying endoscopy. There was no report allowing a strict assessment of the add-on effect of concurrent use of EUS and magnifying endoscopy. Eventually, the recommendation statement reads “there is weak evidence to suggest that EUS or magnifying endoscopy may be useful.” Meanwhile, non-magnifying endoscopy magnifying endoscopy and EUS are widely used as healthcare services covered by the national health insurance program in Japan, and concurrent use of these procedures would entail no problem in that they are inexpensive and minimally invasive modalities.

Most of the studies represented retrospective analyses of prospective data, while only 1 study represented a prospective investigation in the strict sense of the term. Many of the studies were judged to a high risk of bias in the study quality evaluation performed using Quality Assessment of Diagnostic Accuracy Studies (QUADAS). The strength of evidence was thus rated as C.

Workup by EUS and magnifying endoscopy is covered by the national health insurance. Therefore, taking into consideration the benefit–risk balance, strength of evidence and patient preferences, we concluded that “there is weak evidence to recommend ultrasound endoscopy or magnifying endoscopy for clinical diagnostic differentiation between T1a-EP/LPM and T1a-MM disease among cases of superficial cancer of the esophagus.”

#### CQ5: Is assessment of the circumferential extent recommended for esophageal cancer lesions that are selected for endoscopic treatment based on the depth of invasion of the tumor?

### Recommendation statement

There is strong evidence to recommend assessment of the circumferential extent of the lesion prior to the start of treatment for esophageal cancer lesions that are selected for endoscopic treatment on the basis of the depth of invasion of the *tumor (Rate of consensus: 100% [20/20], strength of evidence: A*).

### Explanatory note

It is empirically recognized that there is a strong potential for the esophageal lumen to become narrowed due to scar contraction following endoscopic treatment of esophageal cancer lesions of a large diameter, and it is stated in the Guidelines for Diagnosis and Treatment of Carcinoma of the Esophagus 2nd Edition published in 2007, that only lesions covering ≤ 2/3rd of the circumference constitute absolute indications for endoscopic treatment.

A search of the literature in relation with this CQ yielded 87 PubMed articles and 96 ICHUSHI articles. These articles were subjected to a primary and secondary screenings for original articles related to endoscopic treatment, and finally, 3 papers dealing with observational investigations were subjected to qualitative and quantitative systematic reviews.

Katada et al. reported that postoperative stenosis occurred in 13 of 216 lesions of esophageal cancer treated by endoscopic mucosal resection (EMR) and that the treatment had consisted of a > 3/4th circumferential resection [15]. Ono and associates reported the occurrence of postoperative stenosis in 5 of 6 patients treated by endoscopic submucosal dissection (ESD), in whom the circumferential extent of the lesion exceeded 3/4th of the esophageal circumference [16]. A report by Shi et al. described that postoperative stenosis occurred in 32 of 34 patients with esophageal cancer treated by ESD in whom the circumferential extent of the lesion exceeded 3/4th of the esophageal circumference [17].

Meta-analysis of the data from these 3 papers revealed that the risk ratio for the development of stenosis following endoscopic treatment was 30.93 [95% confidence interval (CI) 18.85–50.76] (*p* < 0.001) for lesions involving > 3/4th of the esophageal circumference as compared to lesions involving ≤ 3/4th of the esophageal circumference.

It is important to predict the risk of postoperative stenosis. Assessment of the circumferential extent of the tumor by endoscopy is covered by the national health insurance, and is neither expensive nor time-consuming. Therefore, taking into consideration the benefit–risk balance, strength of evidence and patient preferences, we have concluded the following: there is strong evidence to recommend assessment of the circumferential extent of the lesion prior to the start of treatment for esophageal cancer lesions that are selected for endoscopic treatment on the basis of the depth of invasion of the tumor.

#### CQ6: What would be recommended for preventing postoperative stenosis following endoscopic treatment of esophageal cancer?

### Recommendation statement

There is strong evidence to recommend any one of the prophylactic balloon dilatation, local steroid injection, or oral steroid administration for the prevention of stenosis after endoscopic treatment (*Rate of consensus: 90% [18/20], strength of evidence: A*).

### Explanatory note

As stated under the Recommendation for CQ5, some or other measures to prevent stenosis are desired, inasmuch as endoscopic treatment of esophageal cancers with the circumferential extent of the lesion exceeding 3/4th of the esophageal circumference entails a high risk of postoperative stenosis [15–17].

A search of the literature in relation with this CQ yielded 122 PubMed articles and 61 ICHUSHI articles. These articles were subjected to primary and secondary screenings for original articles relating to endoscopic treatment. Finally, 1 paper pertaining to accumulated cases and 4 papers dealing with observational investigations were subjected to a systematic qualitative review.

Inoue et al. reported that they were able to successfully avoid stenosis in all 6 patients who underwent ESD for a fully encircling lesion of the esophagus, by repeated prophylactic balloon dilatation from the early phase after the operation. Similarly, Ezoe et al. reported a significantly lower incidence of stenosis in 29 patients with esophageal cancer who underwent endoscopic resection lesions involving > 3/4th of the esophageal circumference and received prophylactic balloon dilatation begun within 1 week after the operation, as compared to a group that did not receive prophylactic balloon dilatation [18].

On the other hand, Hashimoto et al. reported that postoperative stenosis was significantly less frequent, and the frequency of balloon dilation required postoperatively was also significantly lower in a group of 21 patients who underwent subtotal circumferential resection and received postoperative submucosal triamcinolone injection, as compared to the non-injected group [19]. Hanaoka et al. also conducted a prospective study in which submucosal triamcinolone injection was given postoperatively to 30 patients who underwent resection of esophageal cancer involving > 3/4th of the esophageal circumference (excluding total circumferential resection cases) and reported a good efficacy of the measure [20]. A study reported by Yamaguchi et al. demonstrated a preventive effect of oral prednisolone treatment (at the starting dose 30 mg/day, followed by tapering of the dose, for 8 weeks) in 19 patients with esophageal cancer who underwent subtotal-to-total circumferential resection [21].

There is no report, however, of comparative assessment to determine which of the three aforementioned prophylactic measures might show superior efficacy in preventing stenosis. A study aimed at prospective comparative evaluation of the stenosis-preventive effect of submucosal triamcinolone injection and oral prednisolone treatment (JCOG1217) is ongoing and its results are yet to be presented. There are no reports as yet of comparative studies using a combination of stenosis-prevention measures. While balloon dilatation is the only method currently covered by the national health insurance, local steroid injection and oral steroid administration are added to the review herein, as they are relatively inexpensive and safe measures that can be applied in the clinical practice setting.

It is considered more beneficial to adopt stenosis-prevention measures than to undertake esophageal dilatation after the symptoms of stenosis have already developed. Therefore, it is strongly recommended that any one of the prophylactic balloon dilatation, local steroid injection, or oral steroid administration be adopted in patients undergoing endoscopic treatment for lesions involving > 3/4th of the esophageal circumference. With regard to the incidence rate of complications, nevertheless, there are no reports of any systematic reviews; patients should be given sufficient explanation concerning the risk of complications, such as intraoperative perforation, following prophylactic balloon dilation, late-stage perforation following local steroid injection, and systemic infection following oral steroid administration.

#### CQ7: Which is recommended, chemoradiotherapy or radiotherapy, in patients with cStage I esophageal cancer who are unsuitable candidates for surgical treatment?

### Recommendation statement

There is strong evidence to recommend chemoradiotherapy in patients with cStage I esophageal cancer who are unsuitable candidates for endoscopic resection (*Rate of consensus: 84.2% [16/19], strength of evidence: C*).

### Explanatory note

A search of the literature in relation with this CQ yielded 108 PubMed articles, 18 Cochrane articles, and 48 ICHUSHI articles. These articles, together with 6 other articles considered adequate to fulfill the criteria for screening, were subjected to primary and secondary screenings. Finally, 10 papers were subjected to a qualitative systematic review. There was no published randomized comparative study of radiotherapy or chemoradiotherapy solely in cStage I cases. A report of a randomized comparative study in cases with other disease stages and cases of adenocarcinoma [22] and 2 articles dealing with systematic reviews [23, 24] were also retrieved. Two articles representing single-group prospective studies of chemoradiotherapy in patients with cStage I esophageal cancer [25, 26] and a paper on a single-group prospective study of radiotherapy for cStage I or II cancer patients aged ≥ 80 years [27] were also retrieved. There were also 4 articles based on retrospective cohort studies in patients with cStage I disease only (2 articles of studies involving intergroup comparison, and 2 articles based on single-group radiotherapy studies) [28–30].

Cooper et al. conducted a randomized comparative study of radiotherapy and chemoradiotherapy in patients with T1-3 N0-1 M0 esophageal cancer [22]. The study was non-randomized in part, and the 5-year survival rate was 0% in the radiotherapy alone group and 26% in the randomized chemoradiotherapy group. During the course of follow-up, 21% of patients from the randomized chemoradiotherapy group remained recurrence free. The incidence rate of Grade 4 adverse events was 2% in the radiotherapy alone group, while it was as high as 8% in the randomized chemoradiotherapy group. Both systematic reviews of 2 studies of patients not limited to cStage I, including the above-cited study, revealed superiority of chemoradiotherapy to radiotherapy in respect of the survival time and duration of recurrence-free survival [23, 24]. One systematic review including adverse event analysis showed a greater incidence of adverse events in patients who received chemoradiotherapy than in those who received radiotherapy (hazard ratio for Grade ≥3 adverse events during the acute phase: 5.16) [24].

A prospective phase II clinical study (the JCOG9708 Study) conducted in Japan revealed promising results of chemoradiotherapy (60 Gy, cisplatin + 5-fluorouracil [5-FU]) in cStage I cases, with a complete response rate of 87.5%, 4-year survival rate of 80.5%, and 4-year recurrence-free survival rate of 68.1%, with no occurrences of any Grade ≥ 4 adverse events [26]. A gratifying 5-year survival rate (66.4%) was also reported from another prospective chemoradiotherapy (55–66 Gy, cisplatin + 5-FU) plus intracavitary brachytherapy (10–12 Gy) trial [25]. No significant difference in the survival rate was demonstrated in 2 retrospective cohort studies comparing chemoradiotherapy and radiotherapy in cStage I esophageal cancer patients [28]. The reported 5-year survival rates were 50.4–58.7% in 2 retrospective studies of radiation monotherapy [29, 30]. The above 4 retrospective cohort studies included only small numbers of cases (*N* = 36–68) and no adjustments for background factors.

To sum up, although an increase in the incidence of Grade ≥ 3 adverse events was noted in the systematic review of studies not limited to cStage I patients, which was also seemingly within acceptable limits, the significantly longer survival time in patients administered chemoradiotherapy than in those administered radiotherapy and the higher response rate in the former treatment group demonstrated in the JCOG9708 and other studies in patients with cStage I disease lead to the recommendation of chemoradiotherapy rather than radiotherapy alone for the treatment of cStage I esophageal cancer.

In elderly patients with cStage I esophageal cancer who are at a high risk for complications and cannot tolerate surgery and patients with depressed visceral functions, nevertheless, the benefit–risk balance should be fully assessed.

Taking into consideration the benefit–risk balance, strength of evidence and patient preferences, the recommendation statement reads as follows: there is strong evidence to recommend chemoradiotherapy in patients with cStage I esophageal cancer who are unsuitable candidates for surgery/endoscopic resection.

## Treatment algorithm for cStage II–III esophageal cancer (Fig. 2)

### Summary

To select the treatment policy for cStage II and III esophageal carcinoma, the tolerability to surgical intervention should first be confirmed through evaluation of the patient’s general condition after accurate diagnosis of the clinical stage by means of upper gastrointestinal endoscopy, CT, and PET. When no problem is identified with respect to the tolerability for surgical operation, preoperative chemotherapy should be administered, followed by radical resection, as the first-line therapy. Radical resection without preoperative treatment or with preoperative chemoradiotherapy may also be selected. In cases of surgery without any preoperative treatments, administration of adjuvant chemotherapy should be considered in accordance with the histopathologic diagnosis confirmed from resected specimens (especially for lymph node metastasis-positive cases). Definitive chemoradiotherapy (≥ 50 Gy) should be considered in patients who are unable to tolerate surgery, or refuse surgery, in whom chemoradiotherapy is feasible. Patients in whom complete response is achieved should be followed up, and in case of a remnant or recurrent lesion, the practicability of surgical resection as salvage therapy should be explored. In patients unable to tolerate surgery in whom chemoradiotherapy is not indicated either, radiation therapy (e.g., in patients with depressed renal function, elderly subjects), chemotherapy (e.g., in patients with a history of radiation), palliative symptomatic treatment, or palliative chemotherapy should be considered.

**Fig. 2:**
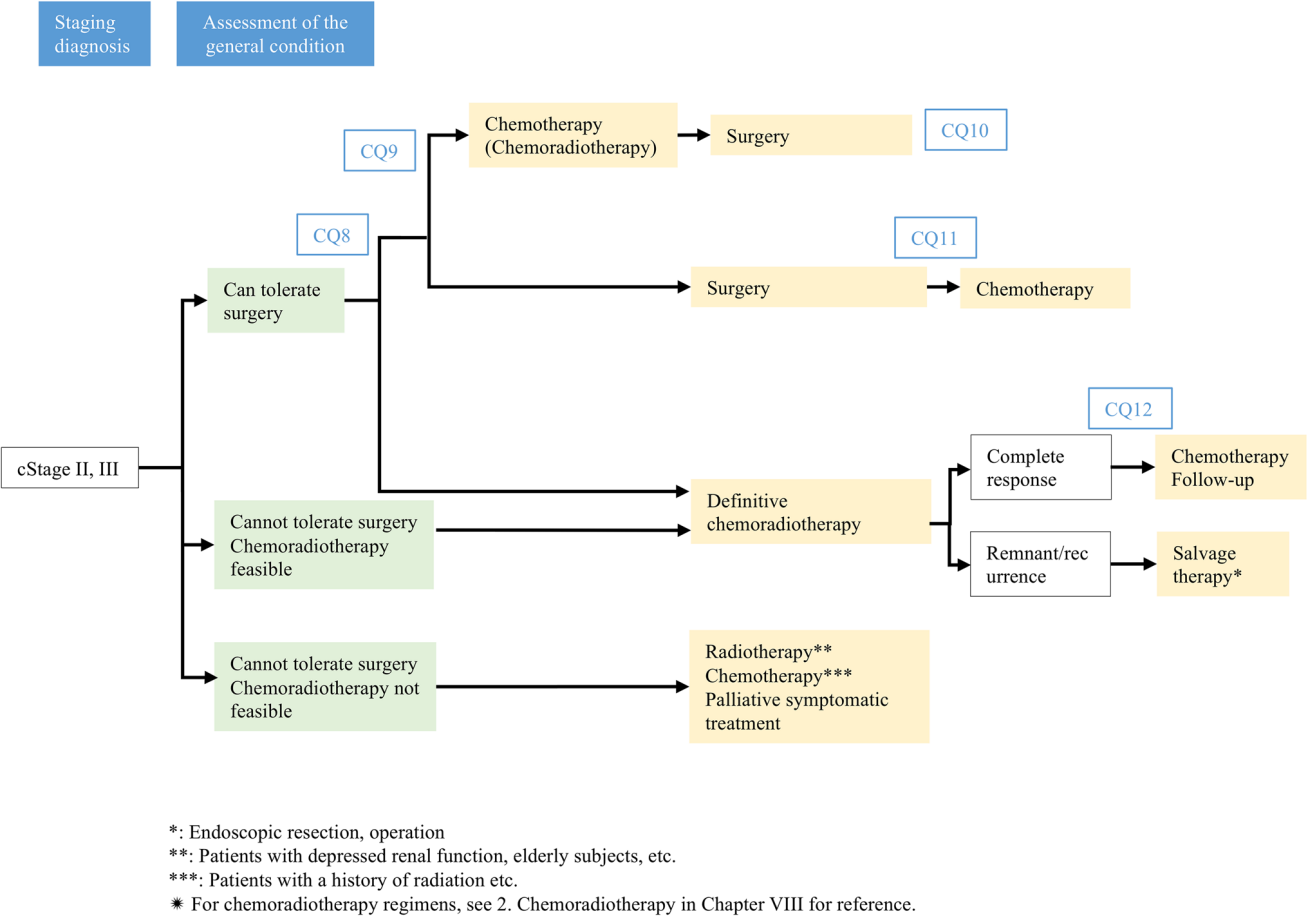
Treatment algorithms for cStage II, III esophageal cancer

#### CQ8: Which is recommended, therapy primarily consisting of surgery or definitive chemoradiotherapy, in patients with cStage II or III esophageal cancer?

### Recommendation statement

There is weak evidence to recommend therapy primarily consisting of surgery for patients with cStage II or III esophageal cancer (*Rate of consensus: 70% [14/20], strength of evidence: C*).

### Explanatory note

For the treatment of cStage II or III esophageal cancer, preoperative chemotherapy + surgery is recommended in view of the results of the JCOG9907 Study [31]. Meanwhile, definitive chemoradiotherapy is also one of the treatment alternatives that offer the potential for radical cure.

A search of the literature in relation with this CQ yielded 486 PubMed articles, 306 Cochrane articles, and 167 ICHUSHI articles, which were subjected to primary and secondary screenings. Finally, 3 papers dealing with randomized comparative studies and 11 papers related to observational studies were retrieved and subjected to a qualitative systematic review.

There are 3 reports of randomized comparative studies directly comparing the results of surgery and definitive chemoradiotherapy [32–34]. Because all these reports are from overseas, however, they differed greatly in the therapy regimens and treatment policies adopted as compared to those in Japan.

In regard to the results of definitive chemoradiotherapy in Japan, the 5-year survival rate was 36.8% in the JCOG9906 Study, which was a single-group phase II clinical study [35].

With regard to observational studies, 10 papers of studies comparing surgery and definitive chemoradiotherapy in patients with cStage II or III esophageal cancer, including 6 papers from Japan, were retrieved [36–44]. None of the studies was a randomized comparative study; therefore, many of the studies differed in terms of the background factors of the patients and in the therapeutic regimens adopted from those currently adopted in Japan. As for comparison of the survival time, 3 of the 10 papers indicated that surgery yielded a significant prolongation of the overall survival time. Only 1 paper indicated prolongation of the overall survival time in patients administered radical chemoradiotherapy.

Thus, it was rather difficult to draw a conclusion to respond to this CQ on the ground of the evidence from the systematic review.

With regard to toxicity, the late toxicities reported in patients receiving definitive chemoradiotherapy in the JCOG9906 Study were esophagitis (Grades 3–4; 13%), pericardial effusion (Grades 3–4; 16%), pleural effusion (Grades 3–4; 9%), and radiation pneumonitis (Grades 3–4; 4%); death of 4 cases was also reported. Out of the 10 observational studies, 6 papers from Japan reported operation-related deaths at an incidence rate of 0–4% among patients treated by surgery. In the JCOG9907 Study, there were 2 cases of operation-related death among 330 cases. It should be noted that there is a potential risk of serious adverse events among patients receiving definitive chemoradiotherapy as well as among those treated by surgery.

The basis for considering that surgery may yield greater improvement of the overall survival rate as compared to definitive chemoradiotherapy is thus rather tenuous, and both treatment modalities entail a significant risk of toxicity. However, there is weak evidence to recommend preoperative chemotherapy plus surgery for the treatment of cStage II or III esophageal cancer, because the 5-year survival rate obtained with this treatment in the JCOG9907 Study was 55%, as compared to 37% in the JCOG9906 Study [45], and because many single-institution observational studies conducted in Japan have revealed more gratifying results in surgically treated groups.

Furthermore, the JCOG0909 Study aimed at assessing the usefulness of preoperative definitive chemoradiotherapy followed by positive surgical intervention as salvage operation in patients with cStage II or III esophageal cancer is now in progress. In patients administered definitive chemoradiotherapy, the benefits and risks of subsequent salvage operation for remnant or recurrent lesions should also be considered. According to a report by Tachimori et al. the incidence of postoperative complications was higher in patients who underwent salvage esophagectomy after definitive chemoradiotherapy at a total dose of 60 Gy, along with an increased in-hospital postoperative mortality of 8%, as compared to 2% associated with surgery, in general [46]. In the JCOG0909 Study, a three-dimensional treatment plan and multiple-field irradiation were introduced and the fractional and total radiation doses were modified to 1.8 and 50.4 Gy, respectively, in an attempt to reduce the risk of adverse events and the risk associated with salvage operation that was seen in the JCOG9906 Study. Accumulation of patients has already been completed and the observation period is now progressing in the study; the results of the study are anticipated for clarification of the usefulness of multimodal treatment, such as definitive chemoradiotherapy plus surgery.

Therapy primarily consisting of surgery and definitive chemoradiotherapy are both covered by the national health insurance, and taking into consideration the benefit–risk balance, strength of evidence and patient preferences, we concluded to state that there is weak evidence to recommend therapy primarily consisting of surgery for patients with cStage II or III esophageal cancer.

#### CQ9: Which of the following three is recommended, preoperative chemotherapy, postoperative chemotherapy, or preoperative chemoradiotherapy, in cStage II or III esophageal cancer patients scheduled to receive therapy primarily consisting of surgery?

### Recommendation statement

In patients with cStage II or III esophageal cancer who are scheduled to receive therapy primarily consisting of surgery:

1. There is strong evidence to recommend preoperative chemotherapy over postoperative chemotherapy (*Rate of consensus: 89.5% [17/19], strength of evidence: B*).
2. There is weak evidence to recommend preoperative chemotherapy over preoperative chemoradiotherapy (*Rate of consensus: 100% [18/18], strength of evidence: C*).

### Explanatory note

A search of the literature in relation with this CQ yielded 419 PubMed articles, 321 Cochrane articles, 98 ICHUSHI articles, which were subjected to primary and secondary screenings with an additional 4 articles, followed by qualitative and quantitative systematic reviews of the selected articles.

In regard to comparison between preoperative chemotherapy and postoperative chemotherapy, the most appropriate timing of adjuvant chemotherapy with cisplatin and 5-FU was assessed in the JCOG9907 Study, whose results showed a significantly better overall survival time in the preoperative chemotherapy group as compared to the postoperative chemotherapy group. There was no difference in the incidence of postoperative complications between the 2 patient groups [47]. Based on these results, preoperative chemotherapy with cisplatin and 5-FU is strongly recommended and defined as a standard therapy for cStage II or III esophageal cancer [31].

Comparison between preoperative chemotherapy and preoperative chemoradiotherapy was then pursued. There was only one article related to a randomized study by Stahl et al. [48], in which preoperative chemotherapy and preoperative chemoradiotherapy were compared in patients with adenocarcinoma of the esophagogastric junction. The study was censored due to poor accumulation of cases, therefore, failing to show any significant difference in the endpoint (overall survival time), although the 3-year survival rate was significantly prolonged in the preoperative chemoradiotherapy group as compared to the preoperative chemotherapy group, indicating the possible usefulness of preoperative chemoradiotherapy. However, the target disorder was adenocarcinoma of the esophagogastric junction in this study, hence differing from the subject patient group referred to in this CQ; therefore, the results were not considered adequate for recommending preoperative chemoradiotherapy as the standard therapy in Japan at present.

On account of the paucity of evidence for arriving at any conclusion in respect of comparison between preoperative chemotherapy and preoperative chemoradiotherapy, we compared the outcomes of preoperative chemoradiotherapy and surgery alone. There has been no randomized comparative study investigating the significance of preoperative chemoradiotherapy in Japan, whereas in Europe and North America, a number of randomized comparative studies examining the usefulness of surgery alone, in view of the limitation in its ability to provide local control, have been reported since latter half of the 1980s [49–61]. The CROSS trial reported by Shapiro et al. [61], comparing a preoperative chemoradiotherapy group and a surgery alone group, demonstrated a significant prolongation of the overall survival time in the former group, in which there was a conspicuous prognostic add-on effect of preoperative chemoradiotherapy, particularly for patients with squamous cell carcinoma. A significant improvement of the postoperative survival rate in the preoperative chemoradiotherapy group was shown by a meta-analysis of data comparing preoperative chemotherapy/chemoradiotherapy and surgery, and surgery alone reported by Sjoquist et al. [62].

Four papers on randomized comparative studies investigating the 5-year survival rate as an outcome [58–61] from among 13 papers dealing with randomized comparative studies of preoperative chemoradiotherapy versus surgery alone conducted in Europe and North America [49–61] were subjected to a qualitative systematic review and meta-analysis. The results revealed no significant intergroup difference in the 5-year survival rate between the two treatment groups, although a tendency towards prolongation of the survival was seen in the preoperative chemoradiotherapy group. The patient groups in the 4 randomized comparative studies differed in part from the population under question in this CQ, in that they also included cStage I and cStage IV cases and also patients receiving carboplatin or paclitaxel or cisplatin alone as chemotherapy.

As regards the toxicities associated with preoperative treatment, according to Kumagai et al. [63], a meta-analysis revealed no increase in the mortality attributable to any type of preoperative treatment in esophageal cancer patients overall, when preoperative chemotherapy and preoperative chemoradiotherapy were compared with surgery alone. However, the report describes an increase in the postoperative mortality and incidence of treatment-related death in the preoperative chemoradiotherapy group as compared to the surgery alone group when the analysis was limited to patients with esophageal squamous cell carcinoma. According to a report by Klevebro et al. [64], the postoperative mortality and incidence of complications were significantly higher in patients receiving preoperative chemoradiotherapy as compared to those receiving preoperative chemotherapy.

While the current standard treatment in Japan is preoperative chemotherapy with cisplatin and 5-FU, evidence suggests that preoperative chemoradiotherapy is also useful. The JCOG1109 Study, as a randomized study to compare the current standard treatment of preoperative chemotherapy with cisplatin and 5-FU and a 3-drug combined preoperative chemotherapy regimen with the addition of docetaxel and preoperative chemoradiotherapy is currently in progress, and results of the study are anticipated [65].

Both preoperative chemotherapy and preoperative chemoradiotherapy are covered by the national health insurance, and considering, based on the benefit–risk balance, strength of evidence and patient preferences, that in patients with cStage II or III esophageal cancer who are scheduled to receive therapy primarily consisting of surgery:

1. There is strong evidence to recommend preoperative chemotherapy over postoperative chemotherapy.
2. There is weak evidence to recommend preoperative chemotherapy over preoperative chemoradiotherapy

#### CQ10: Is postoperative adjuvant therapy recommended in cStage II or III esophageal cancer patients who have undergone preoperative adjuvant therapy plus surgery?

### Recommendation statement

There is weak evidence to recommend against postoperative chemotherapy in cStage II or III thoracic esophageal squamous cell carcinoma patients who have undergone preoperative adjuvant therapy plus surgery (*Rate of consensus: 85% [17/20], strength of evidence: D*).

### Explanatory note

Preoperative chemotherapy plus surgery is currently the standard treatment for cStage II or III esophageal cancer in Japan, as, while surgery plus postoperative chemotherapy was demonstrated to be superior to surgery alone in the JCOG9204 Study [66], the superiority of preoperative chemotherapy to postoperative chemotherapy was demonstrated in the JCOG9907 Study [31]. However, the usefulness of postoperative chemotherapy in patients who have undergone preoperative chemotherapy plus surgery has not yet been sufficiently verified.

A search of the literature in relation with this CQ yielded 315 PubMed articles, 188 Cochrane articles, and 633 ICHUSHI articles through a primary screening, from which secondary screening led to the retrieval of 1 paper dealing with a randomized comparative study [67] and 1 paper dealing with a case–control study [68], which were subjected to a systematic review.

There has been no randomized comparative study published in this regard in Japan, and the 1 paper dealing with a randomized comparative study that was retrieved was from overseas. That study involved comparison of a group of patients with resectable squamous cell carcinoma of the esophagus who received postoperative adjuvant chemotherapy after preoperative chemotherapy plus radical surgery (group A: 175 patients) and a matched group not receiving the postoperative adjuvant therapy (group B: 171 patients), with assessment of the recurrence-free survival time as the primary endpoint. The 5-year recurrence-free survival rate was 35.0% in group A and 19.1% in group B, with a hazard ratio of 0.62 (*p* < 0.001) [67]. However, the surgical procedures and chemotherapy described in the report differ from those used in Japan, and furthermore, the report contains no description of the preoperative staging of the disease; therefore, we consider that the results of this study are not directly applicable to the clinical practice setting in Japan. Meanwhile, pre- and postoperative chemotherapy is undertaken for the treatment of adenocarcinoma in Europe and North America [69, 70].

In general, the completion rate of postoperative chemotherapy is low owing to the high incidence rate of adverse events [31, 69, 70]; therefore, it cannot be concluded at present that the benefits from postoperative chemotherapy outweigh the risks.

While postoperative chemotherapy is covered by the national health insurance, we have concluded, after taking into consideration the benefit–risk balance, strength of evidence and patient preferences, there is weak evidence to recommend against postoperative chemotherapy in cStage II or III thoracic esophageal squamous cell carcinoma patients who have undergone preoperative adjuvant therapy plus surgery.

#### CQ11: Is postoperative chemotherapy recommended for cStage II or III esophageal cancer patients who have undergone surgery without preoperative therapy?

### Recommendation statement

There is only weak evidence to recommend postoperative chemotherapy for cStage II or III esophageal carcinoma patients with pathologically confirmed lymph node metastasis who have undergone surgery without preoperative therapy (*Rate of consensus: 85% [17/20]; strength of evidence: C*).

### Explanatory note

In Japan, radical surgery after preoperative chemotherapy with cisplatin plus 5-FU is recommended for cStage II or III thoracic esophageal cancer patients, on the basis of data from the JCOG9907 Study. In the clinical practice setting, however, surgery alone or surgery plus postoperative chemotherapy is undertaken for these patients, depending on the patient’s condition, in patients who are practically unable to ingest food due to stenosis or any factor that interferes with chemotherapy. There may be cases, where surgery is undertaken under the diagnosis of cStage I disease, but the disease stage is discovered to be cStage II or III; the need for postoperative chemotherapy should be examined in such cases.

A search of the literature in relation with this CQ yielded 260 PubMed articles, 258 Cochrane articles, and 132 ICHUSHI articles, which were subjected to a primary screening. After secondary screening, 3 reports of randomized comparative studies were subjected to qualitative and quantitative systematic reviews [66, 71, 72]. All of these 3 randomized comparative studies entailed a low risk of bias and were consistent. However, the study populations included pStage IV cases and cases in which vindesine was used in the postoperative chemotherapy regimen. The results revealed no appreciable improvement of the 5-year survival rate with postoperative chemotherapy in any of these randomized comparative studies, and a meta-analysis of the 3 randomized comparative studies also yielded the same results [73].

In the JCOG8806 Study, in which the outcomes were compared between a group that received 2 cycles of postoperative cisplatin + vindesine chemotherapy and a surgery alone group, no significant difference in the 5-year survival rate was observed between the two groups, nor was there any add-on effect of postoperative chemotherapy on the survival rate [71]. In the subsequently conducted JCOG9204 Study, in which the outcomes were compared between a group that received 2 cycles of postoperative cisplatin + 5-FU chemotherapy and a surgery alone group, there was no significant difference in the overall survival rate between the two groups, but a significant prolongation of the 5-year recurrence-free survival time was noted in the former group. The prolongation of the recurrence-free survival time was particularly evident in the pathologically lymph node-positive cases [66], and not observed in the pathologically lymph node-negative cases. In a randomized study of postoperative chemotherapy (comparing the outcomes between a group that received 6–8 cycles of postoperative cisplatin + 5-FU chemotherapy and a surgery alone group) conducted in France, only palliative resection was indicated in about a half of the study population, and there was no significant difference in the median survival time between the two groups, indicating the failure of the postoperative cisplatin + 5-FU chemotherapy to prolong the survival [72]. Meta-analysis of the data from these 3 randomized comparative studies revealed a risk ratio of 0.95 (0.78–1.15) (*p *= 0.59), failing to indicate any add-on effect of postoperative chemotherapy on the survival rate.

The long-term outcomes of surgery alone in the JCOG clinical studies conducted until date largely surpass those of the surgery + adjuvant therapy reported from Europe and North America; this trend seems to reflect the substantial differences in the viewpoints about lymphadenectomy and in the precision of lymph node dissection between Japan and Europe/North America. This is an issue that requires attention when comparing the clinical study results between Japan and Europe/North America.

Thus, there is no basis to believe that postoperative chemotherapy would bring about improvement of the overall survival rate in patients undergoing curative resection. Postoperative chemotherapy involves treatment-related death, although at a low incidence, and a definite risk of adverse events, as compared to surgery alone; there was 1 case (0.8%) of treatment-related death among the 120 patients enrolled in the JCOG9204 Study. In the same study, anaemia (1.7%), leukopenia (4.2%), granulocytopenia (15.8%), platelet count decreased (2.5%), nausea/vomiting (8.3%), and diarrhoea (2.5%) were reported as Grade ≥ 3 adverse events, and granulocytopenia (2.5%), arrhythmia (0.8%), infection (0.8%) and pyrexia (0.8%) were reported as Grade ≥4 adverse events. Nevertheless, the study also revealed a significant improvement of the recurrence-free survival rate and prolongation of the recurrence-free survival time in the group that received postoperative chemotherapy, particularly in patients with lymph node metastasis confirmed by histopathology. Based on the results reported from Japan, postoperative chemotherapy (cisplatin + 5-FU, 2 cycles) after curative resection (with no preoperative chemotherapy) is considered to be potentially useful for prolongation of the postoperative recurrence-free survival time in lymph node metastasis-positive patients.

While postoperative chemotherapy (cisplatin + 5-FU, 2 cycles) is covered by the national health insurance in Japan, taking into account the benefit–risk balance, strength of evidence and patient preferences, we concluded that there is only weak evidence to recommend postoperative chemotherapy for cStage II or III esophageal carcinoma patients with pathologically confirmed lymph node metastasis who have undergone surgery without preoperative chemotherapy.

#### CQ12: Is additional chemotherapy recommended for cStage II, III, or IVa esophageal cancer patients who show complete response after chemoradiotherapy?

### Recommendation statement

There is only weak evidence to recommend additional chemotherapy could be offered to cStage II, III, or IVa esophageal carcinoma patients who show complete response after radical chemoradiotherapy (Rate of consensus: 90% [18/20]; evidence level: C).

### Explanatory note

A search of the literature in relation with this CQ yielded 351 PubMed articles, 22 Cochrane articles, and 144 ICHUSHI articles, along with one additional article. Twenty-five papers were extracted through a primary screening, and four papers were extracted after the secondary screening [22, 35, 74, 75]. There was no report of any study comparing additional chemotherapy vs. follow-up observation in patients showing complete response to chemoradiotherapy. Therefore, we carried out qualitative systematic reviews of the 4 extracted reports of large-scale studies of definitive chemoradiotherapy.

In all 4 studies, the chemoradiotherapy consisted of radiation therapy and concurrent chemotherapy, followed by 2 additional cycles of chemotherapy (cisplatin + 5-FU). In 2 of the studies conducted in Japan, the therapeutic response was rated prior to the additional chemotherapy, the latter given only when the patient showed partial response or complete response to the concurrent chemoradiotherapy. Although no clear evidence was presented, it could be assumed that the incidence of adverse events would increase with additional chemotherapy.

There is no evidence to support additional chemotherapy for patients showing complete response to concurrent chemoradiotherapy, and the significance of such therapy has not been clarified. In past large-scale clinical studies of current chemoradiotherapy, however, 2 cycles of additional chemotherapy were included and are generally recognized as an international standard. Nevertheless, careful consideration should be given, because the risks may outweigh the benefits depending on the patient’s condition. Two cycles of additional chemotherapy are covered by the national health insurance.

Thus, taking into account the benefit–risk balance, strength of evidence, and patient preferences, there is only weak evidence to recommend additional chemotherapy could be offered to cStage II, III, or IVa esophageal carcinoma patients who show complete response to radical chemoradiotherapy.

## Treatment algorithm for cStage IV esophageal cancer (Fig. 3)

### Summary

In determining the treatment policy for cStage IV esophageal cancer, assessment of the performance status (PS) is important, besides accurate clinical staging by such means as CT, upper gastrointestinal endoscopy, and PET, as for patients with other clinical stages of the disease.

**Fig. 3:**
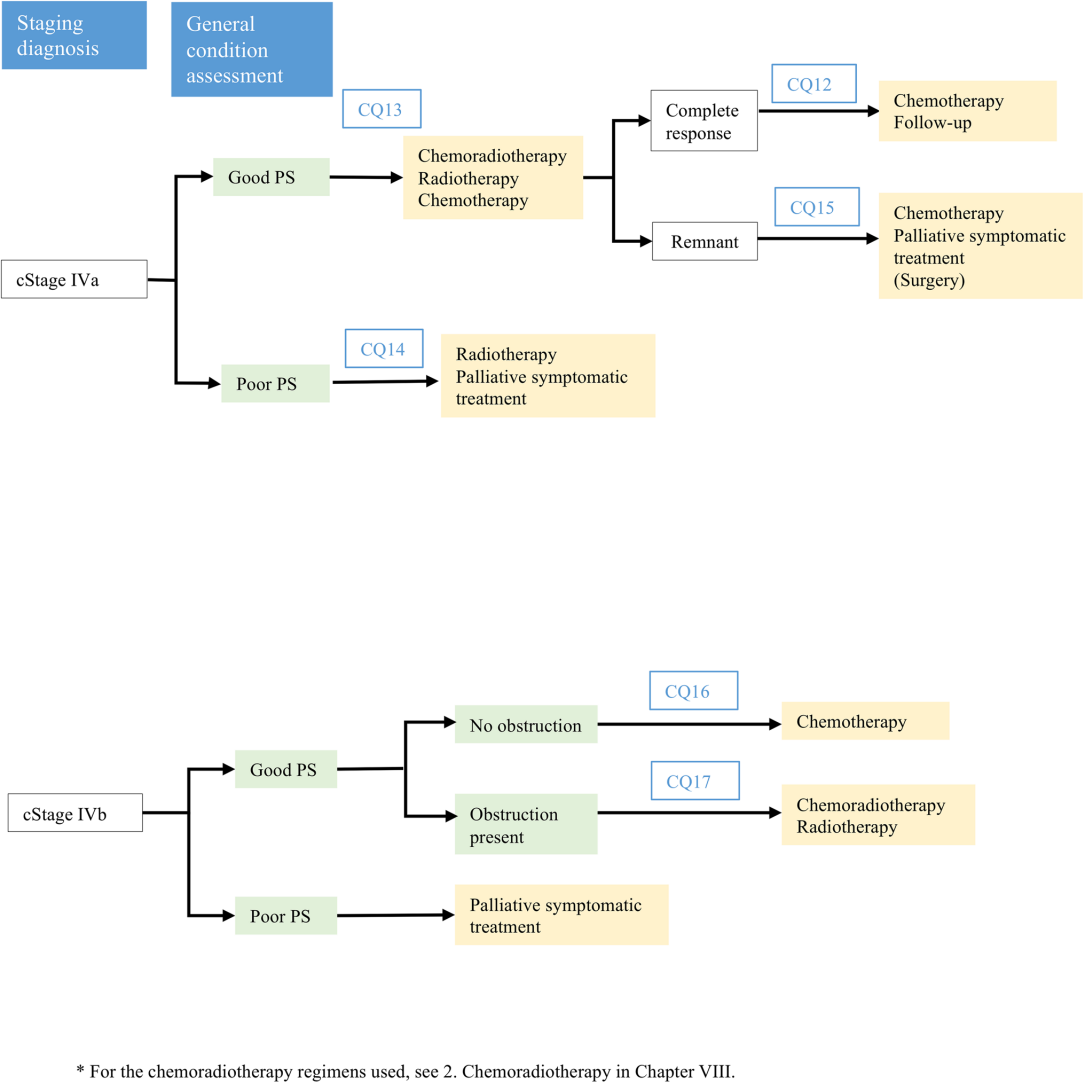
Treatment algorithms for cStage IV esophageal cancer

In patients with cStage IVa cancer with a good PS, definitive chemoradiotherapy is the treatment of choice that may be expected to provide cure. However, the possible need of salvage surgery for local residual lesions after chemoradiotherapy may entail an increase in the risk of operation-related death; therefore, it is necessary to judge the situation comprehensively with due consideration given to the benefit–risk balance. Chemotherapy constitutes the mainstay of treatment for cases with cStage IVb esophageal cancer, which represents progression of the cancer beyond local disease and requires systemic treatment; however, palliative radiotherapy may also need to be considered in patients presenting with evidence of obstruction.

In patients with a poor PS, on the other hand, the main approach is palliative symptomatic treatment. In cases of cStage IVa esophageal cancer, nevertheless, radiotherapy is known to be effective for improving dysphagia caused by the cancer and long-term survival has been reported, although it would still entail a definite risk of adverse events; it represents one choice of treatment.

#### CQ13: Is chemoradiotherapy recommended for cStage IVa esophageal cancer?

### Recommendation statement

There is only weak evidence to recommend radical chemoradiotherapy for treatment of cStage IVa esophageal cancer **(***Rate of consensus: 85% [17/20]; strength of evidence: C*).

### Explanatory note

A search of the literature in relation with this CQ yielded 204 PubMed articles, 114 Cochrane articles, and 145 ICHUSHI articles, along with one additional article. Forty-nine papers were extracted through a primary screening. Six papers extracted through a secondary screening were subjected to a qualitative systematic review. Of these 6 reports, 1 was of a somewhat old and low-quality randomized comparative study pertinent to this CQ, and the other 5 were of studies of definitive chemoradiotherapy.

Definitive chemoradiotherapy is one of the treatment options that could offer an opportunity for cure in patients with unresectable, locally advanced esophageal cancer. However, fatal complications (e.g., tumor perforation, penetration) may also occur owing to a favorable response to chemoradiotherapy. In Japan, in view of the results of the JCOG0303 Study, definitive chemoradiotherapy is often selected for the treatment of patients with unresectable, locally advanced esophageal cancer with a good PS. The validity of this treatment modality was examined by comparison of the percentage of cases showing long-term survival (merit) and the incidence of fatal complications (demerit).

Although there is a paucity of data on the long-term survival, the 2- or 3-year survival rate is reported to be in the range of about 20–30% [76–78], and the percentage of patients showing long-term survival is estimated to be about 15–20%. The patient populations in these studies included a certain proportion of patients with a PS score of 2, and a common feature of these studies was that the prognosis was unfavorable in patients with factors related to a poor PS, such as “weight loss relative to the usual weight,” suggesting the possibility that a significant proportion of the long-term survivors were those with a good PS. Meanwhile, fatal complications (perforation, penetration) were encountered in about 10–20% of cStage IVa patients [79].

One of the extracted reports pertained to a comparison of radiation alone vs. chemoradiotherapy in patients with unresectable, locally advanced esophageal cancer. This was a somewhat old, low-quality randomized comparative study, and the results showing no significant intergroup difference in the survival time, although it should be noted that the radiation/chemotherapy schedule was greatly different from the currently used schedules. Of the other 5 papers, 3 pertained to studies of chemoradiotherapy [77, 78, 80] and 2 papers pertained to single-group prospective studies of chemoradiotherapy administered after induction chemotherapy [81, 82] (Note: the JCOG0303 Study, a randomized comparative study, is treated as a single-group prospective study in this section, because both treatment groups in this study received chemoradiotherapy).

As for radical chemoradiotherapy for cStage IVa esophageal cancer patients with a good PS, there are no reports of direct comparison with other treatment options (no treatment, radiation alone, or chemotherapy alone), although there is a likelihood of some success in terms of radical cure or long-term survival may be expected with radical chemoradiotherapy. The chemotherapy regimens used in the studies referred to herein were mainly cisplatin + 5-FU, use of which is within the scope of the national health insurance in Japan. However, the risk of fatal complications, with a reported incidence of about 10–20%, may be inevitable with this treatment modality, which should, therefore, be selected only after sufficient discussion between the physician and patient about the merits and demerits of the treatment.

From the above results and taking into account the benefit–risk balance, strength of evidence, and patient preferences, we conclude that there is only weak evidence to recommend definitive chemoradiotherapy for treatment of cStage IVa esophageal cancer.

#### CQ14: Is radiotherapy recommended for cStage IVa esophageal cancer in patients with a poor PS?

### Recommendation statement

There is only weak evidence to recommend radiotherapy for the treatment of cStage IVa esophageal cancer patients with a poor PS **(***Rate of consensus: 95% [19/20]; strength of evidence: D*).

### Explanatory note

A search of the literature in relation with this CQ yielded 386 PubMed articles, 150 ICHUSHI articles, and 139 Cochrane articles. Thirty-eight papers were retrieved through primary screening, and 4 papers were extracted through secondary screening, which were then subjected to a qualitative systematic review.

There were 2 reports of studies of chemoradiotherapy for cStage IVa cancer patients, one conducted as a single-center phase II clinical trial and the other as a multicenter phase II clinical trial. Both these studies were conducted in Japan. The treatment schedule consisted of radiotherapy at 60 Gy/30 Fr with concurrent cisplatin + 5-FU chemotherapy (after a 2-week interval). The single-center study conducted in 54 patients reported a response rate of 87%, median survival time of 9 months, and 2-year overall survival rate of 23%, with some toxicities, including Grade ≥ 3 leukopenia (24%) and a platelet count decreased (28%), along with perforation (9%) [75]. The multicenter study conducted in 60 patients reported a response rate of 68%, median survival time of 305.5 days, and 2-year overall survival rate of 31.5%; Grade ≥ 4 toxicities occurred in 8.3% of cases, and the treatment-related death rate was 3.4% [78]. The results were gratifying in respect of the post-treatment survival in both studies, although there was a significant incidence of adverse events in both. However, the significance of chemoradiotherapy in patients with a poor PS in relation with this CQ remains unclear, inasmuch as the details were unknown or were there few patients with a poor PS. In addition, it is generally difficult to administer chemoradiotherapy in patients with a poor PS.

Of 2 comparative studies of intracavitary brachytherapy, one compared two intracavitary brachytherapy schedules [83], while the other explored whether external radiation might be added to intracavitary brachytherapy [84]. In both studies, a survival rate (with a restored ability for swallowing at 6 months after treatment) of ≥50% (median: 7 months approx.) was observed, indicating a gratifying palliative effect. The significance of intracavitary brachytherapy in patients with a poor PS remains unknown, nevertheless, because the study populations in these two studies consisted of patients with a PS of up to 2. In the comparative study comparing two intracavitary brachytherapy schedules, multivariate analysis identified PS as a significant factor influencing the survival rate (with restoration of the ability to swallow) [83]. In Japan, however, intracavitary brachytherapy is scarcely performed, so these results are not directly relevant to the CQ.

To sum up, while there are reports demonstrating the efficacy of chemoradiotherapy and radiation therapy for cStage IVa esophageal cancer, the PS scores of the patients were unknown or only patients with a good PS were included, or they pertained to intracavitary brachytherapy, which is scarcely adopted in Japan; hence, evidence for efficacy is not compelling. Meanwhile, radiotherapy has been shown to be effective for improving dysphagia in esophageal cancer patients, and long-term survivors have been reported, although there is a definite risk of adverse events. It is often the case in clinical practice settings, that patients desire treatments that can yield long-term survival. This treatment is entirely covered by the national health insurance.

Thus, taking into account the benefit–risk balance, strength of evidence, and patient preferences, we concluded that there is only weak evidence to recommend radiotherapy for the treatment of cStage IVa esophageal cancer patients with a poor PS.

#### CQ15: Is surgical treatment recommended for cStage IVa esophageal cancer patients with residual disease after chemoradiotherapy?

### Recommendation statement

There is weak evidence not to recommend surgery in cStage IVa esophageal cancer patients showing residual disease after chemoradiotherapy (*Rate of consensus: 85% [17/20]; strength of evidence: D*).

### Explanatory note

A search of the literature in relation with this CQ yielded 290 PubMed articles, 27 Cochrane articles, and 117 ICHUSHI articles, along with additional 2 other papers. Forty-two papers were retrieved through primary screening, and 2 papers were extracted through secondary screening and subjected to a qualitative systematic review.

The usefulness of additional surgical treatment for cStage IVa esophageal cancer patients whose condition was initially not considered as an indication for surgical resection, who had undergone definitive chemoradiotherapy with a favorable response and feasible resectability of residual disease, was explored. However, there were no reports of comparison of surgical treatment with non-surgical treatment after definitive chemoradiotherapy. The therapeutic outcomes reported in the 2 papers pertaining to surgical therapy after radical chemoradiotherapy were compared with those in studies of mainly non-surgical therapies conducted in Japan.

Both the extracted papers pertained to retrospective observational studies, in which cStage IVa esophageal cancer patients (mostly T4b cases) received surgical therapy after definitive chemoradiotherapy (≥ 50 Gy). According to one of these reports from Italy, 96.1% of 51 patients underwent surgical therapy, and R0 resection was achieved in 39.2% patients. The prognosis tended to be better in the R0 resection cases than in the R1/2 resection cases; the median overall survival time was 11.1 months, the 3-year survival rate was 8.8%, and the 5-year survival rate was 5.9% [85], and the operation-related mortality was 10.2%. In the other study reported from Japan, of 37 patients overall, patients showing clinical complete response underwent follow-up observation, and surgery was performed only in the 13 patients who showed response to chemoradiotherapy. Of the 13 patients, R0 resection was achieved in 12 patients [86]. As in the report from Italy, the prognosis tended to be better in the cases with R0 resection than in those of R1/2 resection; the median overall survival time was 10.1 months, the 1-year survival rate was 45%, the 2-year survival rate was 35%, and the 5-year survival rate was 23%. There was no description about operation-related deaths in this paper.

The current standard treatment for cStage IVa esophageal cancer in Japan, based on the data of the JCOG0303 Study, is standard chemoradiotherapy using cisplatin plus 5-FU (cisplatin 70 mg/m^2^ on day 1, and 5-FU 700 mg/m^2^ on days 1–4; 2 cycles at intervals of 4 weeks; 60 Gy/30 Fr). Of the 71 patients who received standard chemoradiotherapy in the JCOG0303 Study, 12 (approx. 17%) underwent further surgery for residual/recurrent disease after completion of the protocol-specified treatment [77]. The median overall survival time was 13 months, the 1-year survival rate was 56.8%, and the 3-year survival rate was 27.6%. The prognosis of the patients who failed to show complete response was unfavorable. Surgical therapy aimed at R0 resection is also performed in the daily practice setting. Meanwhile, as there is a possibility of increase in the risk of operation-related death, it is important to make the judgment with due consideration given to the benefit–risk balance when surgical therapy is considered.

The intervention relevant to this CQ of surgical therapy is covered by the national health insurance.

For comparing the therapeutic outcome in the JCOG0303 Study with the outcomes in the 2 above-cited papers, it would be necessary to consider the following points: (1) a considerable proportion of the patients treated in the JCOG0303 Study, being a clinical trial, were in a more stable disease state, and (2) the 2 above-cited papers are old data that differ from the current status in terms of both the results of surgery and choices of treatment available for patients with recurrent disease. Nevertheless, it is a fact that surgery after definitive radiation is highly invasive and entails the risks of postoperative complications and treatment-related death. Considering the lack of sufficient ground at present to suggest that surgical intervention can improve the patient prognosis or quality of life (QOL), and taking into account the benefits and risks, we concluded that there is weak evidence not to recommend surgery in cStage IVa esophageal cancer patients showing residual disease after chemoradiotherapy. Currently, a study to compare definitive chemoradiotherapy, being the standard treatment, and a treatment modality consisting of potent induction chemotherapy followed by resection of lesions that have become resectable (JCOG1510 Study) is planned.

#### CQ16: Is chemotherapy recommended for the treatment of cStage IVb esophageal cancer?

### Recommendation statement

There is only weak evidence to recommend chemotherapy for the treatment of cStage IVb esophageal cancer **(***Rate of consensus: 85% [17/20]; strength of evidence: C*).

### Explanatory note

A search of the literature in relation with this CQ yielded 401 PubMed articles, 372 Cochrane articles, and 76 ICHUSHI articles, along with one additional paper. Forty-three papers were retrieved through primary screening, and 41 papers were extracted through secondary screening. Among the 41 papers, there were 3 reports of randomized comparative trials pertinent to this CQ and 35 reports of studies of intervention by chemotherapy and assessable for the benefits and risks. These studies were subjected to a qualitative systematic review.

There was only 1 report of a randomized comparative study of an untreated group vs. chemotherapy group, i.e., of placebo vs. gefitinib [87]. The study failed to show superiority of gefitinib over placebo. In regard to phase II clinical studies using other drugs, primary treatment with a combination of cisplatin plus 5-FU yielded a response rate of about 30% and a median survival time of 6.6–9.5 months [88–91], which led to combined regimens becoming recognized as the standard treatment. Combined treatment with 5-FU and nedaplatin, as an alternative to cisplatin, was also assessed in a phase II study, with the results showing a response rate of about 9.5% and a median survival time of 8.8 months, so that nedaplatin has become a drug of choice in patients in whom cisplatin cannot be used due to renal function and/or cardiac function [92]. All these therapy regimens are covered by the national health insurance of Japan.

With regard to secondary treatment, treatment with paclitaxel 100 mg/m^2^ weekly for 6 weeks, repeated at 7-week intervals, produced gratifying results, with a response rate of 44.2% and median survival time of 10.4 months [93]. With docetaxel alone at 70 mg/m^2^ once every 3 weeks, the response rate was 16% but the median survival time was less at 8.1 months [94]. Adverse events were within a permissible range in patients with a good PS, while Grade ≥ 3 serious adverse events were encountered at an incidence of about 10–20% among patients receiving multi-drug therapy; therefore, caution should be exercised even in patients with a good PS. It is important to make an accurate judgment about whether the treatment can be continued or not, taking into consideration the patient’s quality of life, in patients who develop adverse events affecting quality of life, such as neuropathy and taste disorder.

Mainly patients with PS scores of 0–1 and well-maintained visceral functions were enrolled in these clinical studies. It would reasonable to assume that a certain level of efficacy would be attained in such subjects, although there was no clear comparative study. There exists no evidence to support chemotherapy in subjects with a poor PS; chemotherapy is not recommended, inasmuch as there are no grounds to suggest the efficacy of chemotherapy at present. In patients with a poor PS, palliative symptomatic treatment should be administered first, and if improvement is noted, the patient may then be considered for chemotherapy. Careful consideration must be given to the benefit–risk balance.

Thus, taking into account the benefit–risk balance, strength of evidence, and patient preferences, we concluded that there is only weak evidence to recommend chemotherapy for the treatment of cStage IVb esophageal cancer.

#### CQ17: Is palliative radiotherapy recommended for the treatment of cStage IVb esophageal cancer in patients presenting with obstruction?

### Recommendation statement

There is only weak evidence to recommend palliative radiotherapy for the treatment of cStage IVb esophageal cancer in patients presenting with obstruction (*Rate of consensus: 100% [20/20]; strength of evidence: C*).

### Explanatory note

A search of the literature in relation with this CQ yielded 302 PubMed articles, 46 ICHUSHI articles, and 79 Cochrane articles, from which 29 papers were extracted through primary screening, and, 3 papers and 1 abstract were extracted through secondary screening and subjected to a qualitative systematic review.

According to a report of the results of chemoradiotherapy performed for palliation in cStage IVb esophageal cancer, 40 patients with PS scores of ≤ 2 (≤ 1 in 38 of the 40 patients) suffering from dysphagia received chemoradiotherapy with a cisplatin + 5-FU regimen and 40 Gy/20 Fr radiation, which yielded improvement of the swallowing function score in 75% of the patients. Hematotoxicities were noted, although the incidence remained within permissible range, the median survival time was 308 days, and the 1-year survival rate was 45%; thus, a relatively favorable therapeutic outcome was obtained, although esophageal perforation occurred in 5% of patients and 30-day mortality rate after radiation was 5% [95].

A comparative study of radiation monotherapy vs. chemoradiotherapy in cStage IVb patients suffering from dysphagia and up to cStage III patients who were inappropriate candidates for surgery [96] was extracted in an abstract form; it was included in this evaluation as it was a randomized comparative study in which the subjects and interventions were practically pertinent to this CQ. The abstract gave no information regarding the PS in the patients. The study was conducted in 220 patients and compared the outcomes of palliative radiation alone at 35 Gy/15 Fr or 30 Gy/10 Fr and chemoradiotherapy with cisplatin + 5-FU and the same radiation schedule. The percentage of patients who showed improvement of the dysphagia was 68 and 74% in the two groups, respectively, showing no significant intergroup difference, although gastrointestinal toxicities (nausea and vomiting) were significantly more frequent in the chemoradiotherapy group. The median survival time was 203 days in the palliative radiation group and 210 days in the chemoradiotherapy group, again showing no significant intergroup difference.

There were 2 reports of studies comparing the therapeutic responses to radiotherapy and metallic stenting [97, 98]. An earlier improvement of swallowing was observed in the stent group in both reports, but the improvement in swallowing was better sustained in the radiotherapy (intracavitary brachytherapy) group. The contents of these studies were perhaps not very pertinent to this CQ, because intracavitary brachytherapy, which was used in both of these studies, is scarcely applied in Japan.

To sum up, radiotherapy is effective for improvement of dysphagia, and serious adverse reactions are not necessarily common, although some adverse events do occur. Desire for symptomatic amelioration is generally profound in patients with dysphagia and this treatment is covered by the national health insurance. Taking into account the benefit–risk balance, strength of evidence, and patient preferences, we concluded that there is only weak evidence to recommend palliative radiotherapy for the treatment of cStage IVb esophageal cancer in patients presenting with obstruction.
